# Optimization of Thermal Conductivity of Bismaleimide/h-BN Composite Materials Based on Molecular Structure Design

**DOI:** 10.3390/polym17152133

**Published:** 2025-08-03

**Authors:** Weizhuo Li, Run Gu, Xuan Wang, Chenglong Wang, Mingzhe Qu, Xiaoming Wang, Jiahao Shi

**Affiliations:** 1 School of Intelligence and Civil Engineering, Harbin University, Harbin 150076, China; 2 Key Laboratory of Engineering Dielectrics and Application, Ministry of Education Harbin University of Science and Technology, Harbin 150080, China; 3 School of Material Science and Chemical Engineering, Harbin University of Science and Technology, Harbin 150080, China

**Keywords:** bismaleimide, h-BN, thermal conductivity, molecular structure design

## Abstract

With the rapid development of information technology and semiconductor technology, the iteration speed of electronic devices has accelerated in an unprecedented manner, and the market demand for miniaturized, highly integrated, and highly intelligent devices continues to rise. But when these electronic devices operate at high power, the electronic components generate a large amount of integrated heat. Due to the limitations of existing heat dissipation channels, the current heat dissipation performance of electronic packaging materials is struggling to meet practical needs, resulting in heat accumulation and high temperatures inside the equipment, seriously affecting operational stability. For electronic devices that require high energy density and fast signal transmission, improving the heat dissipation capability of electronic packaging materials can significantly enhance their application prospects. In order to improve the thermal conductivity of composite materials, hexagonal boron nitride (h-BN) was selected as the thermal filling material in this paper. The BMI resin was structurally modified through molecular structure design. The results showed that the micro-branched structure and h-BN synergistically improved the thermal conductivity and insulation performance of the composite material, with a thermal conductivity coefficient of 1.51 W/(m·K) and a significant improvement in insulation performance. The core mechanism is the optimization of the dispersion state of h-BN filler in the matrix resin through the free volume in the micro-branched structure, which improves the thermal conductivity of the composite material while maintaining high insulation.

## 1. Introduction

In the era of pursuing high performance and stability, thermal conductive materials have become indispensable key materials in various national defense and economic fields [1,2,3]. From military equipment to consumer electronics, thermally conductive materials are continuously empowering industrial upgrading in microelectronic packaging and component heat dissipation scenarios. With the vigorous rise of 5G, new energy, and intelligent manufacturing, rapid technological development is driving electronic devices to achieve high integration, multifunctionality, and miniaturization upgrades [4,5,6]. The power density of electronic components continues to rise, resulting in a significant increase in heat generation. However, the lag in heat dissipation technology has reduced the operational performance and service life of devices, while also seriously hindering the development of high power and high integration in the field of electronic communication [7,8,9]. Developing electronic packaging materials with high thermal conductivity has become the key to solving this problem. At the same time, how to adopt high thermal conductivity, low cost, and environmentally friendly preparation methods to achieve the large-scale application of materials is still an urgent challenge that needs to be overcome, and requires collaboration between academia and industry [10,11,12].

BMI resin exhibits significant advantages as a new generation of thermal conductive matrix resin due to its excellent heat resistance, insulation performance, and low thermal expansion coefficient [13]. BMI resin possesses the excellent high-temperature resistance properties of polyimide (PI) resin, with a thermal decomposition temperature greater than 420 °C, and excellent high-temperature mechanical, dimensional, and insulation stability, making it widely used in important fields such as electrical insulation, transportation, aerospace, and the military industry [14,15,16]. The low thermal conductivity of BMI, the high viscosity of its resin melt, and the brittleness of the cured materials limit its further application as a thermal conductive material [17].

Common thermal conductive fillers for BMI-based thermally conductive composite materials include hexagonal boron nitride (h-BN), carbon materials, silicon nitride (Si_3_N_4_), aluminum nitride (AlN), and aluminum oxide (Al_2_O_3_) [18,19]. For electronic devices, it is necessary to ensure a high power density while achieving a small size and light weight, which requires thermally conductive composite materials to have a lower density. Si_3_N_4_ and Al_2_O_3_ have a high density (>3.2 g/cm^3^) and hardness, and as thermal conductive fillers, they can significantly increase the density of thermally conductive composite materials. Their high hardness can cause wear and damage to the insulation structure during packaging, leading to insulation damage; AlN has poor storage stability and reacts with water to generate Al(OH)_3_ and NH_3_ in humid environments; carbon fillers have a significant impact on the insulation performance of thermally conductive composite materials and are costly. BN, as a new type of ceramic material with excellent performance and great development potential, and h-BN, as the most commonly used form of boron nitride, has a chemical structure similar to graphite, presenting a hexagonal layered structure. It has extremely high thermal conductivity and insulation strength, and its low density (<2.25 g/cm^3^) is conducive to the production of lightweight electronic devices. Its soft texture is wear-resistant in electronic devices [20,21,22].

For composite thermal conductive materials, when there is a significant density difference between the filler and the matrix resin, gravity will cause free settling, and the rate follows Stokes’ law, which states that increasing particle size, increasing density difference, or decreasing resin viscosity will significantly enhance the settling trend. At the same time, insufficient compatibility between fillers and resins can easily cause particle agglomeration, leading to the formation of large-sized aggregates and further exacerbating sedimentation. If the dispersant in the system is insufficient or the dispersion process is unreasonable, it is difficult for the filler to maintain colloidal suspension stability, ultimately leading to the deposition of particles under the dominance of gravity. This study used molecular structure design to prepare branched polymers and constructed a multi-scale dispersion control system through their unique three-dimensional structure, which facilitates the dispersion of thermal conductive fillers in the polymer. The pore network of the branched skeleton promotes the wetting and permeation of the thermally conductive filler, and the active functional groups in the molecular chain segments form directional chemical bonds with the surface of the filler, synergistically constructing a stable filler matrix interface to optimize the dispersion of the filler and simultaneously improve the thermal conductivity efficiency and insulation performance of the composite material.

Based on the above analysis, this study systematically carries out structural design and functional modification research on a high-performance resin matrix due to the limited heat dissipation performance caused by insufficient heat resistance level, low thermal conductivity coefficient, and other material performance defects in thermal conductive composite materials. Constructing a molecular structure design BMI resin system establishes the relationship between the branched monomer molecular structure and addition amount and the comprehensive electrical thermal properties of the BMI resin; the influence of the content of thermally conductive fillers on the formation mechanism of thermal conductivity pathways in composite materials was systematically studied by filling h-BN to improve the thermal conductivity of the BMI resin.

## 2. Experimental

### 2.1. Materials

Diphenylmethane type bismaleimide (BMI) was purchased from Shandong Minghoude Polymer Materials Co., Ltd., Binzhou, China; ortho diallyl bisphenol A (DBA) was purchased from Shandong Laiyu Chemical Co., Ltd., Laizhou, China; hexagonal boron nitride (h-BN) was purchased from Shanghai Macklin Biochemical Co., Ltd., Shanghai, China; Tert-Butyl peroxybenzoate (TBPB) was purchased from Shanghai Macklin Biochemical Co., Ltd., Shanghai, China; toluene diisocyanate trimer (TDI_3_) was purchased from Shandong Minghoude Polymer Materials Co., Ltd., Binzhou, China; Maleic anhydride (MA) was purchased from Shandong Minghoude Polymer Materials Co., Ltd., Binzhou, China; N,N-dimethylacrylamide (DMAA) was purchased from Jinan Yuyi Chemical Co., Ltd., Jinan, China; N. N-dimethylformamide (DMF) was purchased from Shanghai Macklin Biochemical Co., Ltd., Shanghai, China; sodium bicarbonate (NaHCO_3_) was purchased from Shanghai Macklin Biochemical Co., Ltd., Shanghai, China.

### 2.2. Preparation of BMI-DBA-TTMI/DMAA/h-BN Composite Material

**Figure 1:**
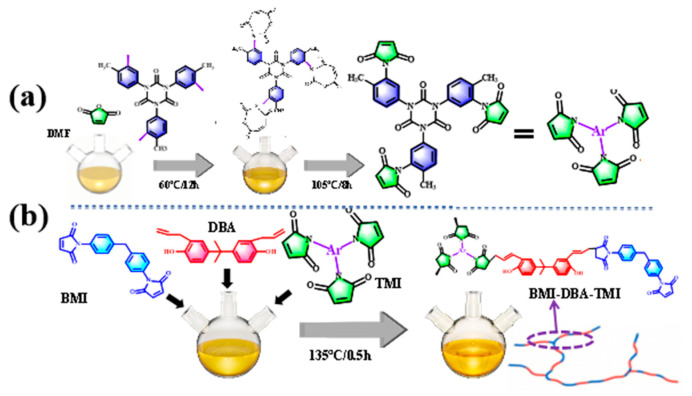
Flowchart for the Preparation of BMI-DBA-TTMI/DMAA: (**a**) Flowchart for the Preparation of TTMI; (**b**) Flowchart for the Preparation of BMI-DBA-TTMI.

Using TDI_3_ as the core and MA as the capped monomer, the mixture was added to DMF in a molar ratio of 1:3.05. The reaction was carried out using a reflux condenser at 60 °C for 12 h, and nitrogen was used for protection throughout the entire reaction process. After the reaction was complete, the temperature was raised to 105 °C, and an appropriate amount of hydroquinone was added as a polymerization inhibitor for a constant temperature reflux reaction for 8 h. Then, a vacuum distillation device was used and the temperature was raised to 110 °C for 2 h to remove DMF. The resulting reaction mixture was washed multiple times with NaHCO_3_ aqueous solution and dried at least three times to obtain the tri-functional maleimide branched monomer TTMI. The preparation process of the branched monomer is shown in Figure 1a.

BMI, DBA, and TTMI massage ratios (1:0.775:0.15) were added to a three-necked flask and heated in a constant temperature 135 °C oil bath for 25 min. After the reaction was completed, the temperature was lowered to 50 °C, and 30 wt% DMAA diluent of the total resin mass was added separately. The mixture was maintained at 50 °C and stirred for 1 h. After stirring evenly, it was poured out into a material bottle to obtain the micro-branched bismaleimide resin solution BMI-DBA-TTMI/DMAA. The preparation process of micro-branched modified BMI resin is shown in Figure 1b [23].

h-BN was added to BMI-DBA-TTMI/DMAA adhesive solution at certain mass fractions (10 wt%, 20 wt%, 30 wt%, and 40 wt%) and dispersed in a high-speed disperser for 6 h to obtain the BMI-DBA-TTMI/DMAA/h-BN composite adhesive solution. Place BMI-DBA-TTMI/DMAA/h-BN and TBPB into a three-necked bottle in a mass ratio of 100:1, and disperse under vacuum in an oil bath at a constant temperature of 80 °C for 30 min. After it is evenly dispersed, pour it into a polytetrafluoroethylene mold and place it in an oven for curing. The curing temperature program is set to 135 °C/2 h + 160 °C/2 h + 200 °C/2 h + 230 °C/4 h.

### 2.3. Characterization

The chemical structure of the resin was characterized using a Fourier transform infrared spectrometer with JASCO 6100 model from Bruker AG, Germany. The test wavenumber range was 4000–500 cm^−1^, and the scanning frequency was set to 32 times.

The microstructure of the cross-section of the composite material sample was observed using the ZEISS GeminiSEM 300 scanning electron microscope from Carl Zeiss in Germany.

The conductivity current was tested using a self-made three-electrode testing system. The test field strength was set to 10 kV/mm, the test frequency was 50 Hz, the test temperature was selected as 25–150 °C, the step size was 25 °C, and each temperature point was maintained for 30 min before measurement. Calculate the AC conductivity using Equation (1) as follows:

*J* = *γ_v_*
*E* (1)

where *E* represents the applied field strength (V/m) for testing, and *J* represents the conductivity current density (A/m^2^). *γ_v_* represents conductivity.

The pressurization method used during the testing process is a uniform pressurization with a pressurization rate of 1 kV/s. Ten effective tests were conducted on the same sample for each measurement, and the relevant data was recorded. Probability statistics were performed using a two-parameter Weibull distribution, and the Weibull distribution failure probability density distribution is calculated using Equation (2)

(2) $$P(E)=1-exp[(\frac{E}{Eb})\beta$$

where P(*E*) is used to represent the cumulative failure probability; *E* represents the breakdown field strength, *Eb* represents the characteristic breakdown field strength obtained by fitting the two-parameter Weibull distribution when the cumulative failure probability is 63.2%, and *β* represents the shape parameter.

The LFA447 laser thermal conductivity meter produced by the German company Netzsch was used to test the thermal conductivity of composite materials. The test sample has a diameter of 12.7 mm and a thickness of 1 mm.

The thermal mechanical properties of the material were tested using the TAQ800 Dynamic Thermomechanical Analyzer (DMA) from TA Instruments in the United States. The test was conducted using a double cantilever beam fixture with a sample size of 40 × 6 × 0.6 mm, a heating rate of 5 °C/min, a frequency of 1 Hz, and a testing temperature of 50–300 °C.

The thermal stability of the cured resin was tested using a TGA2 thermogravimetric analyzer from Mettler, Switzerland. The heating rate was set at 5 °C/min and the heating range was 50–800 °C. The test was conducted in a nitrogen environment with a gas flow rate of 30 mL/min.

## 3. Results and Discussion

### 3.1. Structural Characterization of BMI-DBA-TTMI/DMAA

**Figure 2:**
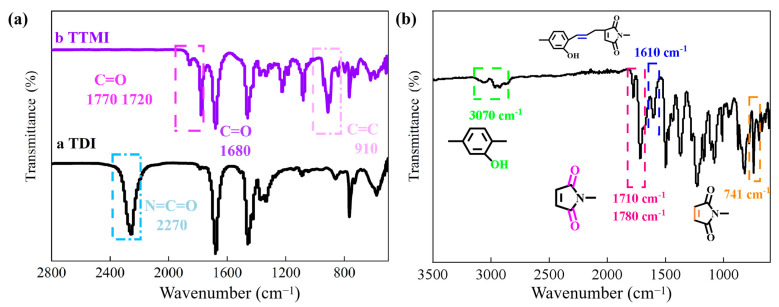
FT-IR of BMI-DBA-TTMI/DMAA. (**a**) TTMI; (**b**) BMI-DBA-TTMI.

Figure 2a shows the infrared characterization of the branched monomer structure TMI. From curve a, it can be seen that the vibration frequency of the N=C=O group can be observed at 2270 cm^−1^, while the absorption peak does not appear in curve b after reacting with MA. In addition, curve b exhibits a C=C absorption peak at 910 cm^−1^. In curve b, the coupling peaks of C=O on the newly generated imine ring are located at 1680 cm^−1^ and 1780 cm^−1^, providing preliminary evidence for the successful synthesis of a TMI-branched monomer structure by an MA-terminated isocyanate trimer.

Figure 2b shows the infrared characterization spectrum of BMI-DBA-TTMI/DMAA. The vibration peaks of the double bond groups in maleimide are all located at 741 cm^−1^, and the coupling bimodal curves induced by C=O on the imide ring are located at 1710 cm^−1^ and 1780 cm^−1^, respectively. The absorption peak of the newly generated C=C after synthesis appears at 1610 cm^−1^ [24,25]. In addition, the absorption peak of the phenolic hydroxyl group in the curve appears at 3560 cm^−1^, and the absorption peak of the phenolic hydroxyl group in the curve appears at 3070 cm^−1^, confirming the successful synthesis of BMI-DBA-TTMI/DMAA.

### 3.2. Microscopic Morphology of BMI-DBA-TTMI/DMAA/h-BN Composite Materials

**Figure 3:**
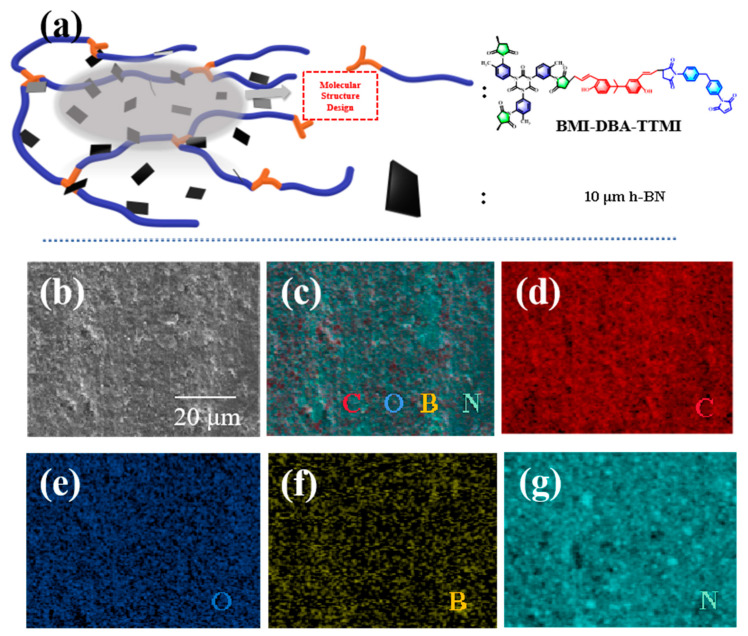
(**a**) Schematic diagram of BMI-DBA-TTMI/DMAA/h-BN composites. (**b**) Sectional SEM, (**c**) mapping diagram, (**d**) C element, (**e**) O element, (**f**) B element, and (**g**) N element.

To verify the dispersibility of h-BN in the resin matrix, the morphology of the cross-section of the BMI-DBA-TTMI/DMAA/h-BN composite material was characterized, and the results are shown in Figure 3. As shown in Figure 3a, the double maleimide resin designed by the molecular structure method has numerous branching points and the molecular chains are difficult to entangle with each other, resulting in the low viscosity of the resin. This makes it have good fluidity during dispersion so it does not form large aggregates, thus maintaining excellent dispersibility. The designed molecular structure of bismaleimide designed by the molecular structure method has a large number of holes, which makes it difficult for the molecular chains to entangle with each other, thereby reducing the interaction between particles and facilitating dispersion. It also has numerous active functional groups at its end, which can form strong interactions with h-BN and enhance its dispersibility in the gel solution. Figure 3b presents the SEM images and meta-mapping images of BMI-DBA-TTMI/DMAA/h-BN composite materials. By observing the energy spectra of B and N elements in Figure 3b, it can be seen that the dispersion of the h-BN thermally conductive filler is relatively uniform, and the phenomenon of filler agglomeration is extremely rare. This means that its dispersibility has been improved, and that molecular structure design can improve the dispersibility of h-BN in the matrix resin.

### 3.3. Thermal Conductivity Characteristics

**Figure 4:**
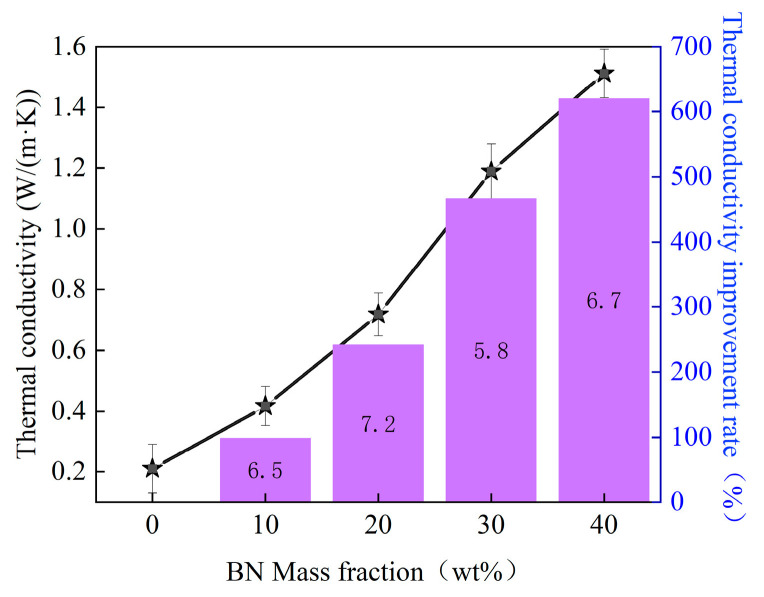
The effect of different h-BN addition contents on the thermal conductivity of BMI-DBA-TTMI/DMAA/h-BN.

To investigate the effect of h-BN addition on the heat transfer capacity of thermally conductive composite materials, the thermal conductivity of BMI-DBA-TTMI/DMAA/h-BN was tested, and a thermal conductivity improvement line graph was drawn based on its thermal conductivity. The data graph is shown in Figure 4.

From Figure 4, it can be seen that the thermal conductivity of BMI-DBA-TTMI/DMAA/h-BN increases with the increase in h-BN addition. When the h-BN addition reaches 40 wt%, the thermal conductivity reaches 1.51 W/(m·K), which is 620% higher than that of BMI-DBA-TTMI/DMAA/h-BN.

**Figure 5:**
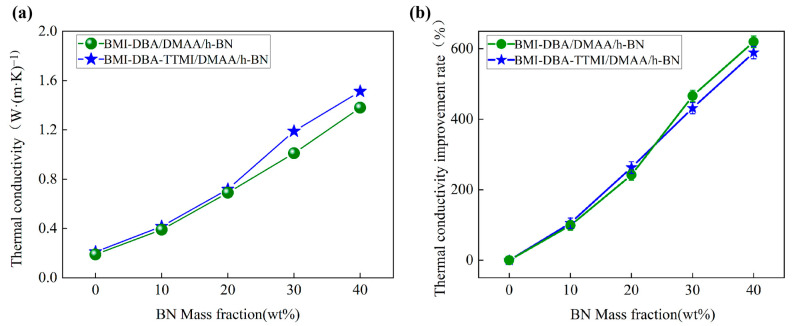
Thermal conductivity diagram of different materials. (**a**) Thermal conductivity; (**b**) Thermal conductivity improvement rate.

In order to compare the effect of molecular structure design on the heat transfer performance of thermal conductive composite materials, Figure 5 summarizes and compares the changes in thermal conductivity and the improvement line chart of thermal conductivity between BMI-DBA/DMAA/h-BN and BMI-DBA-TTMI/DMAA/h-BN. As shown in Figure 5, with the increase in h-BN filling rate, the thermal conductivity of BMI-DBA/DMAA/h-BN increases from 0.19 W/(m·K) to 1.31 W/(m·K), with an improvement rate of 589.47%, while the thermal conductivity of BMI-DBA-TTMI/DMAA/h-BN increases from 0.21 W/(m·K) to 1.51 W/(m·K), with an improvement rate of 620%. Therefore, molecular structure design significantly improves the thermal conductivity of composite materials.

**Figure 6:**
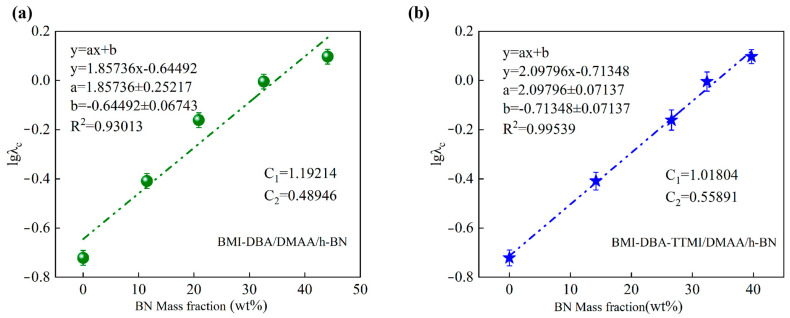
Fitting results of the thermal conductivity model of composite material; Y. Agari. (**a**) BMI-DBA/DMAA/h-BN; (**b**) BMI-DBA-TTMI/DMAA/h-BN.

By conducting thermal conductivity tests on BMI-DBA/DMAA/h-BN and BMI-DBA-TTMI/DMAA/h-BN with filling amounts of 10 wt%, 20 wt%, 30 wt%, and 40 wt%, and constructing a thermal conductivity efficiency model based on the Y. Agari model [26,27], referring to Equation (3), the results are shown in Figure 6.

lg*λ_c_* = *V_f_C*_2_lg*λ_f_* + (1 − *V_f_*) lg (*C*_1_*λ_p_*) (3)

After fitting and calculating the composite material using the Y. Agari model, the results are shown in Table 1. The C_1_ values of BMI-DBA/DMAA/h-BN and BMI-DBA-TTMI/DMAA/h-BN are 1.192 and 1.108, respectively, and the C_2_ values are 0.489 and 0.599, respectively. Relatively speaking, the C_2_ value of BMI-DBA-TTMI/DMAA/h-BN is relatively higher, indicating that h-BN is more likely to construct thermal conductivity pathways or networks in the BMI-DBA-TTMI/DMAA/h-BN resin system. Compared with BMI-DBA/DMAA/h-BN, the C_1_ value of BMI-DBA-TTMI/DMAA/h-BN is closer to 1, indicating that h-BN is more likely to form a thermal conductivity pathway or network within the BMI-DBA-TTMI/DMAA/h-BN resin system.

In order to explore the mechanism by which molecular structure design improves the thermal conductivity of thermally conductive composite materials, according to the EMT model [28], the normalized thermal conductivity is defined as *K_e_*/*K_m_*, where *K_e_* and *K_m_* represent the effective thermal conductivity of the composite material and the matrix resin, respectively. This theory assumes that the filler is ideally distributed in the matrix. In order to better explain the mechanism by which molecular structure design improves the thermal conductivity λ of thermally conductive composite materials, this article also uses the EMT model. The EMT equation is shown in Equation (4)

(4) $$\frac{K_{e}}{K_{m}}=1+\frac{fp\times K_{c}/K_{m}}{3p+\frac{2R_{k}K_{m}}{d}\frac{K_{c}}{K_{m}}}$$

Among them, *f* represents the volume fraction of h-BN filler, *p* = l/d is the aspect ratio of h-BN, where l is the length of h-BN and d is its thickness. *K_c_* and *R_k_* are the thermal conductivity and interfacial thermal resistance of h-BN, respectively.

**Figure 7:**
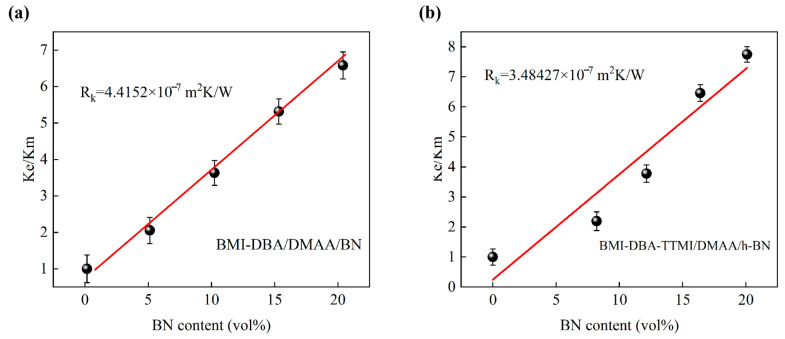
Interfacial thermal resistance fitting between the internal packing and the matrix of the composite material. (**a**) BMI-DBA/DMAA/h-BN; (**b**) BMI-DBA-TTMI/DMAA/h-BN.

In this EMT model, the following parameters were set: *p* = 50, *d* = 200 nm, *K_c_* = 300 W/(m·K), *K_m_* = 0.19 W/(m·K), and *K_e_* = 300 W/(m·K). Based on these parameters for fitting, the calculated *R_k_* value is shown in Figure 7.

The interfacial thermal resistance fitting between the matrix resin and the filler is shown in Figure 7, and the fitting method is linear fitting. The calculation results show that the interface thermal resistance between the internal filler and matrix of BMI-DBA/DMAA/h-BN is 4.42 × 10^−7^ m^2^ K/W, while the interface thermal resistance between the internal filler and matrix of BMI-DBA-TTMI/DMAA/h-BN is 3.48 × 10^−7^ m^2^ K/W. This indicates that molecular structure design can significantly reduce the interfacial thermal resistance between the filler and matrix inside the material and efficiently improve the thermal conductivity efficiency of thermally conductive composite materials. In practical situations, the dispersion state of fillers in the matrix resin often cannot be completely ideal. Therefore, Huang et al. [29,30,31] proposed a modified EMT equation to further analyze the effect of filler dispersion on the thermal conductivity of thermally conductive composite materials. Equation (5) is

(5) $$\frac{K_{e}}{K_{m}}=1+\frac{p\times K_{c}/K_{m}}{3p+\frac{2R_{k}K_{m}}{d}\frac{K_{c}}{K_{m}}}{\left(f-f_{c}\right)}^{t}$$

**Figure 8:**
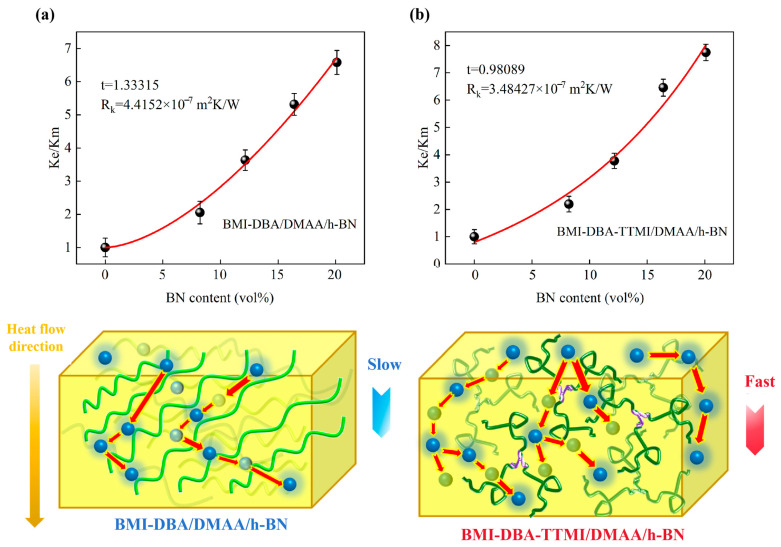
Thermal resistance fitting and thermal conduction network of composite interface after modified EMT model. (**a**) BMI-DBA/DMAA/h-BN; (**b**) BMI-DBA-TTMI/DMAA/h-BN.

This revised equation considers the dispersion state of the filler; specifically, the critical volume fraction *f_c_* of h-BN is represented by 0.6/p, and *t* is the key parameter characterizing the distribution state of the filler. When h-BN is uniformly dispersed, *t* = 1; when *t* > 1, it indicates that h-BN is not well dispersed in the matrix resin. Normally, the smaller the *t*-value, the better the dispersion of the filler. For this purpose, the modified EMT Equation (5) was used for further analysis, and the calculation results are shown in Figure 8.

As shown in Figure 8, the *t*-values of BMI-DBA/DMAA/h-BN and BMI-DBA-TTMI/DMAA/h-BN are 1.33 and 0.98, respectively. This calculation result indicates that after molecular structure design, the *t*-values have decreased, indicating an improvement in the dispersion of h-BN in BMI-DBA-TTMI/DMAA/h-BN. As shown in Figure 8b, due to the “ordered” and “disordered” distribution of h-BN in the resin, for BMI-DBA/DMAA/h-BN, the thermal conductive filler is randomly dispersed inside the BMI resin, resulting in incomplete and discontinuous thermal conduction paths. There is a significant thermal resistance at the interface between “BN-BN” and “BN-BMI”, which limits the improvement of thermal conductivity. Using molecular structure design to create an internal free volume and constructing voids at the end of the molecular chain is beneficial for optimizing the dispersion of h-BN. With the construction of a continuous thermally conductive network, the h-BN within the BMI-DBA-TTMI/DMAA/h-BN resin framework achieves effective overlap, creating a high-speed channel for phonon transmission and thus improving the thermal conductivity of the thermal conductive composite material.

**Figure 9:**
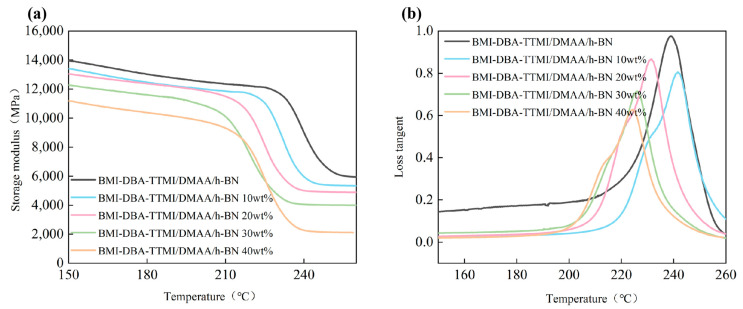
The DMA curves of BMI-DBA-TTMI/DMAA/h-BN. (**a**) Storage modulus; (**b**) Loss tangent.

In order to investigate the relationship between h-BN and the dynamic thermomechanical properties of BMI-DBA-TTMI/DMAA/h-BN, DMA testing was conducted, and the results are shown in Figure 9.

The DMA test results of BMI-DBA-TTMI/DMAA/h-BN with different h-BN contents can be seen from the figure. It can be seen from the graph that as the h-BN content increases, the glass storage modulus and *T*_g_ of the BMI resin-cured material first increase and then decrease. When added to 40 wt%, *T*_g_ decreases to 224 °C. In crosslinked polymer systems with fillers, traditional elastic theory calculates the apparent crosslink density ρ of the cured material through E. However, the shape, stiffness, and interaction between boron nitride and the matrix often lead to an overstatement of the crosslink density calculated by the elastic modulus method, resulting in an overestimation of the actual crosslink density. To solve this problem, the Halpin Tsai model was used for correction [32,33], which effectively separated the enhancement effect of fillers on the modulus of the thermally conductive potting adhesive from the true contribution of cross-linked networks through semi-empirical formulas, thereby reverse-correcting the matrix modulus and eliminating errors caused by the high modulus of the fillers themselves. Calculate the matrix resin Er using Equation (6)

(6) $$E_{r}=\frac{E_{M}}{1+\zeta \eta V}\times (1-\eta V_{f})$$

Here, *E_M_* is the modulus of the cured material measured by DMA, *V_f_* is the volume fraction of the filler, and *ζ* is the shape factor in the model, which describes the influence of filler geometry on the modulus enhancement efficiency of the thermally conductive sealant. For sheet-like boron nitride, the equation for calculating its shape factor is (7)

(7) $$\zeta =2\times \frac{L}{d}$$

Here, *L* is the diameter of the filler sheet, and *d* is the thickness of the filler. In this experiment, a sheet diameter of 10 μm and a thickness of 200 nm were used, and the calculated *ζ* of the sheet-like boron nitride was 100. Here, *η* is a constant, and its calculation equation is as follows (8):

(8) $$\eta =\frac{E_{F}/E_{R}-1}{E_{F}/E_{R}+\zeta }$$

According to literature research, the storage modulus of h-BN is 90 GPa, and the calculated *η* of the composite material is 0.123. Therefore, the *E_R_* was obtained through the Halpin Tsai correction model, and the apparent crosslink density ρ of the cured material containing fillers was calculated to evaluate the relevant parameters of the crosslinking degree of the resin network structure, as shown in Table 2.

From Table 2, it can be seen that the apparent crosslinking density of BMI-DBA-TTMI/DMAA/h-BN gradually decreases with the introduction of h-BN. High-filling h-BN inhibits the movement of resin molecular segments during crosslinking, thereby reducing the degree of material reaction. Pure BMI-DBA-TTMI/DMAA/h-BN resin has a multifunctional structure with multiple crosslinkable points and a high crosslink density, resulting in a high *T*_g_. From Figure 9 it can be seen that the addition of h-BN hinders the collision of molecular segments during crosslinking and curing, thereby reducing the crosslinking density and *T*_g_ of the BMI-DBA-TTMI/DMAA/h-BN-cured material. However, overall, the *T*_g_ change in the BMI-DBA-TTMI/DMAA/h-BN-cured material is not significant, and it has a high *T*_g_.

**Figure 10:**
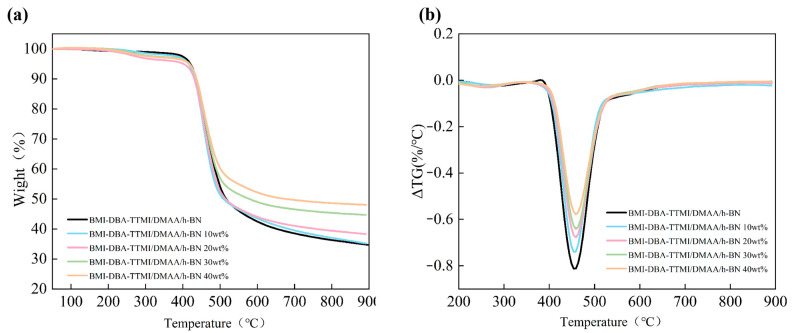
The TGA curves of BMI-DBA-TTMI/DMAA/h-BN. (**a**) TGA; (**b**) DTG.

Figure 10 presents the TGA and DTG curves of BMI-DBA-TTMI/DMAA/h-BN. By comparing the thermal decomposition temperatures T_5%_ of different curves, it can be clearly observed that the T_5%_ of the BMI-DBA-TTMI/DMAA/h-BN sample is 459 °C. This is due to its rich benzene rings and rigid functional groups, such as imide rings, which give it excellent heat resistance. The addition of h-BN resulted in a slight decrease in the thermal decomposition temperature of the material, and samples with a higher h-BN filling content exhibited higher residual mass fractions. Figure 10b shows the DTG curve of BMI-DBA-TTMI/DMAA/h-BN. With the addition of h-BN, the maximum thermal decomposition rate of the material slightly decreases, but the decrease is not significant, indicating that the addition of h-BN slightly reduces the DTG peak of the sample. The research results show that adding an appropriate amount of h-BN particles to BMI-DBA-TTMI/DMAA/h-BN can maintain the thermal stability of the composite material and reduce the quality loss of the sample.

### 3.4. Insulation Properties

**Figure 11:**
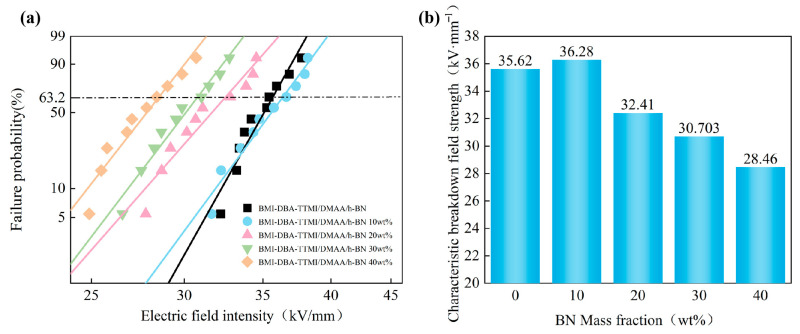
The breakdown strength of BMI-DBA-TTMI/DMAA/h-BN. (**a**) Weibull distribution diagrams; (**b**) Characteristic breakdown field strength.

In order to investigate the effect of the amount of h-BN filling on the insulation performance of thermally conductive composite materials, the AC breakdown field strength of BMI-DBA-TTMI/DMAA/h-BN was tested. Two Weibull distribution diagrams were drawn for analysis, and the breakdown field strength of the composite material is shown in Figure 11. The relevant parameters are shown in Table 3.

As shown in Figure 11, the AC breakdown field strength of BMI-DBA-TTMI/DMAA/h-BN prepared from h-BN with a diameter of 10 μm shows a trend of first increasing and then decreasing with the increase in filling amount. Moreover, the AC breakdown field strength of h-BN composite material is the highest at low filling amounts (10 wt%). Under low-addition conditions, the good interface bonding and synergistic effect formed between h-BN and the BMI-DBA-TTMI/DMAA/h-BN matrix restricts the carrier migration path, effectively improving the electrical strength of the material [34]. In the short-term breakdown process of solid dielectrics, breakdown develops in the form of electrical branches. When the electric branch touches the filler, due to the high electrical strength of h-BN itself, it can only “bypass” or “continue to develop along” the surface of h-BN. This carrier scattering mechanism significantly improves the breakdown field strength. When the filler content exceeds 10 wt%, the insulation performance of the composite system shows a decreasing trend. This is mainly due to the increase in viscosity of the resin system when the filler content is high, resulting in an increase in internal defects of the composite material, and the high addition of thermal conductive components hindering the curing degree of the resin. At the same time, the introduction of a large amount of h-BN will form an “impurity bridge”. In a strong electric field environment, charge carriers can jump between fillers with continuous structures, which also reduces the insulation strength of the composite material to a certain extent.

**Figure 12:**
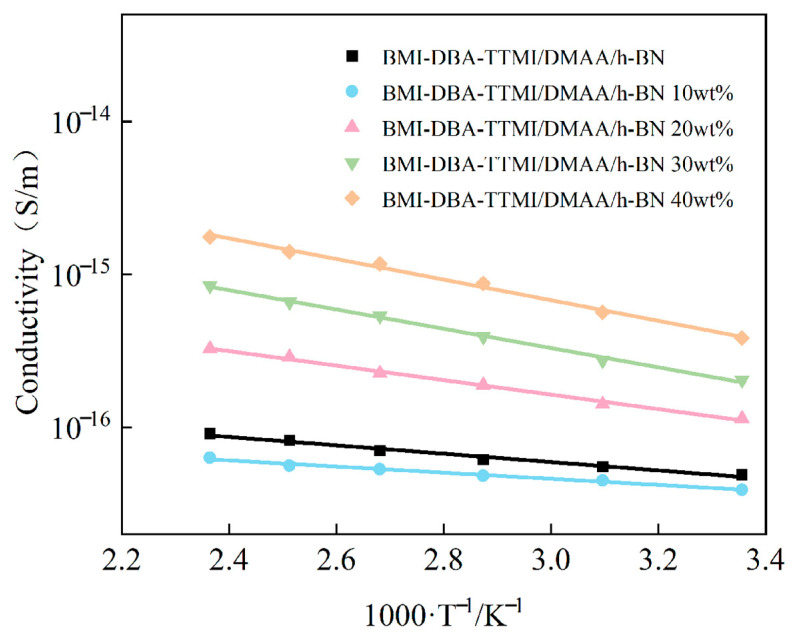
The conductivity of BMI-DBA-TTMI/DMAA/h-BN with the reciprocal of temperature.

The variation in AC conductivity of BMI-DBA-TTMI/DMAA/h-BN with temperature in the temperature range of 25 °C to 150 °C under an electric field strength of 10 kV/mm is shown in Figure 12.

As shown in Figure 12, the conductivity of BMI-DBA-TTMI/DMAA/h-BN still exhibits a linear relationship with the reciprocal of absolute temperature. During the temperature rise, the large molecular chains in BMI-DBA-TTMI/DMAA/h-BN tend to relax, and the amorphous region vibrates strongly. The ability of the BMI-DBA-TTMI/DMAA/h-BN resin matrix to bind to charge carriers weakens, resulting in an increase in charge carrier mobility and a significant increase in the conductivity of the composite material. From Figure 12, it can be seen that the conductivity of BMI-DBA-TTMI/DMAA/h-BN first decreases and then increases. This is because the sheet-like h-BN restricts the migration of charge carriers in the BMI-DBA-TTMI/DMAA/h-BN resin, thereby reducing the mobility of charge carriers in the composite material. However, when the mass fraction of the filler exceeds 10 wt%, due to the hydrogen bonding between h-BN and the hydroxyl group of BMI-DBA-TTMI/DMAA/h-BN, through surface oxygen or nitrogen atoms, excessive hydrogen bonding will reduce the curing degree of BMI-DBA-TTMI/DMAA/h-BN, leaving too many polar functional groups that have not participated in the reaction in the matrix, thereby leading to an increase in the conductivity of the composite material to a certain extent. From Figure 12, it can be observed that when the addition amount is 40 wt%, the sheet-like h-BN has a more significant effect on the conductivity increase in BMI-DBA-TTMI/DMAA/h-BN. This is mainly due to the thickening effect of the smaller h-BN on the resin, the poor mobility of the molecular chain segments resulting in a decrease in crosslinking density, and the appearance of some defects in the cured material, ultimately affecting the insulation performance of the composite material. The introduction of large-sized fillers will reduce the filling rate of the resin, have an adverse effect on the dispersion of the resin, and thus cause the insulation layer to become thinner after molding, hindering the curing reaction of the resin, resulting in internal voids, and causing adverse consequences for the performance of the insulation layer.

To investigate the temperature sensitivity of BMI-DBA-TTMI/DMAA/h-BN conductivity, the test results were fitted to obtain the activation energy E of the material’s conductivity, as shown in Table 4. According to Table 4, the activation energy of composite materials generally shows a trend of first decreasing and then increasing with the increase in h-BN content. This is mainly due to the addition of an appropriate amount of h-BN filler, which increases the number of interfacial layers inside the material. As the temperature increases, the volume of space occupied by the interfacial layer expands, enhancing the scattering ability of charge carriers and increasing the inhibitory effect of the composite material on charge carrier migration. Therefore, in high-temperature environments, the interface layer plays a role in reducing the temperature sensitivity of composite materials [35]. However, excessive addition of h-BN can lead to a decrease in the cross-linking density of BMI-DBA-TTMI/DMAA/h-BN, causing the three-dimensional network structure to become loose and the molecular segments to relax more easily at high temperatures, thereby increasing the sensitivity of the composite material to temperature.

**Figure 13:**
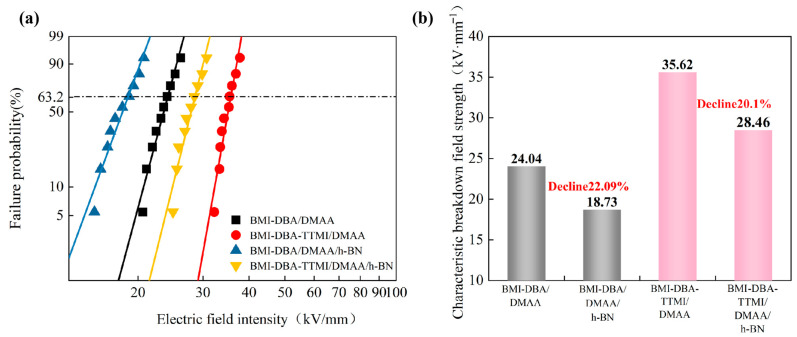
Comparison chart of breakdown strengths of different materials. (**a**) Weibull distribution diagrams; (**b**) Characteristic breakdown field strength.

In order to investigate the effect of molecular structure design on the AC breakdown field strength of materials, Figure 13 compares the changes in the breakdown field strength of the resin and 40 wt% h-BN composite materials filled both before and after molecular design, as shown in Table 5.

From Figure 13, it can be seen that the breakdown field strength of BMI-DBA-TTMI/DMAA is higher than that of BMI-DBA/DMAA. This is because the addition of TTMI greatly improves the crosslinking degree of the BMI resin, resulting in a decrease in carrier mobility under strong electric field conditions, thereby improving the breakdown performance of the cured material. The BMI-DBA resin contains low-molecular-weight DMAA, which reduces the cross-linking degree of the BMI resin, loosens the originally dense cured structure, and easily forms local defects, promoting the migration of charge carriers and increasing the collision ionization of charge carriers. Therefore, the AC breakdown field strength of BMI-DBA-TTMI/DMAA/h-BN is higher. From Figure 13, it can be observed that the AC breakdown field strength of BMI-DBA/DMAA/h-BN is smaller than that of BMI-DBA-TTMI/DMAA/h-BN. This is because the introduction of TTMI is more conducive to the uniform dispersion of h-BN in the resin system, while increasing the cross-linking density of the material, making the bonding between h-BN and the resin tighter, with strong interactions, effectively limiting the movement of charge carriers. The DMAA oligomers in BMI-DBA make the resin structure loose, increasing the mobility of charge carriers. Therefore, the AC breakdown field strength of BMI-DBA-TTMI/DMAA/h-BN is better than that of BMI-DBA/DMAA/h-BN.

**Figure 14:**
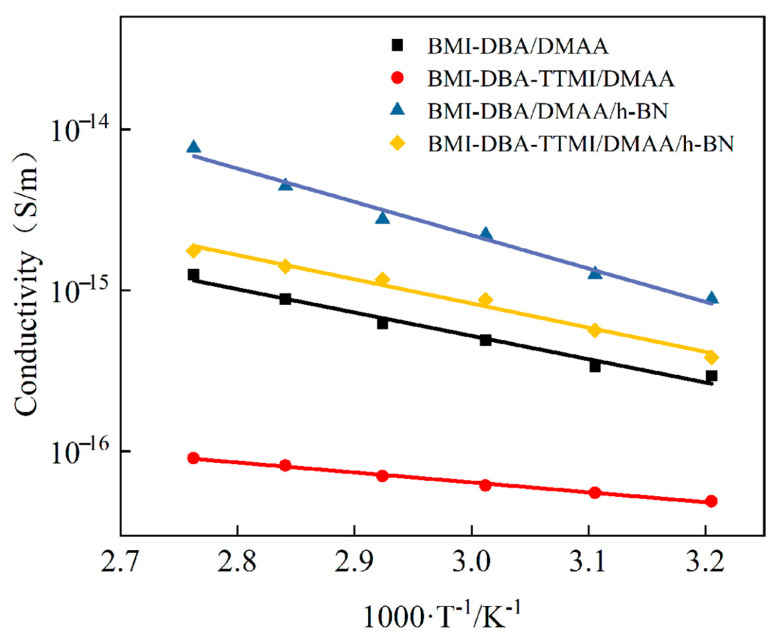
The conductivity of the different materials varies with the reciprocal of the temperature.

In order to investigate the effect of molecular structure design on the AC conductivity of materials, Figure 14 summarizes the changes in the AC conductivity of the resin and composite materials filled with 40 wt% h-BN before and after molecular structure design. From Figure 14, it can be seen that the AC conductivity of BMI-DBA-TTMI/DMAA is higher than that of BMI-DBA/DMAA, indicating that the formation of micro-branched structures is a key factor in changing material properties. The increase in TTMI enhances the cross-linking degree of the BMI resin, and the branching unit structure acts like an “anchoring” function, effectively limiting the activity of chain segments and reducing the probability of carrier migration, thereby lowering the conductivity of the material.

In order to investigate the temperature characteristics of material conductivity, the activation energy E of the material is shown in Table 6. As shown in the table, the activation energy of the material generally decreases with the increase in filler content, mainly due to the increase in crosslinking density caused by TTMI. As the temperature increases, the increase in crosslinking density limits the thermal mobility of the main chain segment of the molecule, making the material’s inhibitory effect on carrier migration more pronounced. Therefore, in high-temperature environments, TTMI plays a role in reducing the material’s sensitivity to temperature. From Figure 14, it can be observed that the AC conductivity of BMI-DBA/DMAA/h-BN is greater than that of BMI-DBA-TTMI/DMAA/h-BN. This is because the introduction of TTMI is more conducive to the uniform dispersion of h-BN in the resin system, making the bonding between h-BN and the resin tighter, while increasing the cross-linking density of the material, effectively limiting the movement of charge carriers. The DMAA oligomers in BMI-DBA make the resin structure looser, increasing the mobility of charge carriers. Therefore, the improvement of the insulation performance of composite materials by micro-branched structures is mainly achieved through two aspects: Firstly, the molecular segments themselves contain high FFV, and their unique three-dimensional structure enhances the intermolecular interaction force. The hydroxyl and other polar functional groups in the segments have strong adsorption effects on the thermally conductive powder, which can effectively disperse these particles and maintain their stable dispersion state. This is conducive to the formation of a good organic-inorganic interface, with more and more uniform thermal conductive filler resin interfaces, and stronger restriction on charge carriers. Secondly, the micro-branched structure can increase the crosslinking density of the resin, and its branched units act as anchors, strongly restricting the movement of molecular segments. Under the action of an electric field, it effectively reduces the mobility of charge carriers in the composite material and maintains high insulation performance at high temperatures.

**Figure 15:**
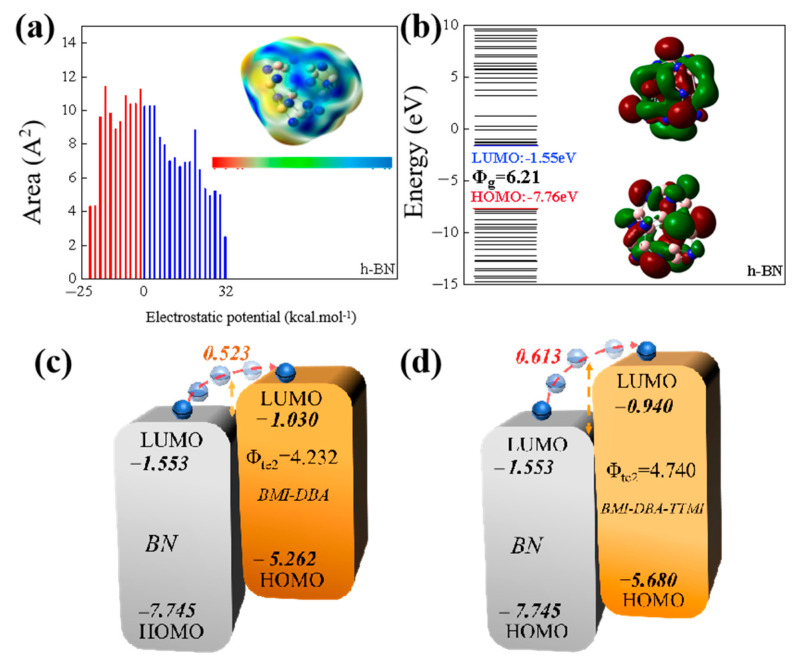
The electrostatic potential and energy and distribution diagrams of different materials. (**a**) Electrostatic potential; (**b**) Energy level distribution; (**c**) Electron hopping barrier of BMI-DBA/DMAA/h-BN; (**d**) Electron hopping barrier of BMI-DBA-TTMI/DMAA/h-BN.

By calculating the electrostatic potential distribution and energy level orbitals of h-BN, it can be seen that h-BN has the ability to attract electrons and holes. Figure 15a,b shows the electrostatic potential and energy level distribution of h-BN molecules. From the distribution map of electrostatic potential, it can be seen that h-BN occupies 73.6% of the area in the positive electrostatic potential region, indicating that h-BN has strong electronegativity. From the analysis of the band structure, it can be seen that the HOMO orbital level of h-BN is −7.66 eV, the LUMO orbital level is −1.55 eV, and the band gap is 6.21 eV, indicating that h-BN has a larger bandgap, making it more difficult for electrons to migrate from the valence band to the conduction band. As shown in Figure 15c,d, the HOMO orbital level of BMI-DBA is −5.262 eV, the LUMO orbital level is −1.030 eV, and the band gap is 4.23 eV, while the HOMO orbital level of BMI-DBA-TTMI is −5.680 eV, the LUMO orbital level is −0.940 eV, and the bandgap width is 4.74 eV. The BMI-DBA-TTM structure significantly increases its bandgap width, thereby increasing the potential barrier difference between h-BN and the matrix resin in terms of LUMO energy levels. As shown in Figure 15c,d, the electron hopping barrier of BMI-DBA/DMAA/h-BN is 0.523 eV, while the barrier difference required for electron hopping in BMI-DBA-TTMI/DMAA/h-BN increases to 0.613 eV. A high barrier can weaken the injection of charge carriers, while h-BN has a scattering effect on electrons, which synergistically reduces the electron mobility. Therefore, appropriate h-BN improves the insulation performance of the composite material, and the overall insulation performance of BMI-DBA-TTMI/DMAA/h-BN is higher than that of BMI-DBA/DMAA/h-BN.

The molecular structure design significantly improves the comprehensive performance of the thermal conductivity composite, and at the same time, the insulation performance is also improved. This collaborative optimization effect originates from the optimization mechanism of molecular structure design on the interface of composite materials. By enhancing the dispersion of fillers through a branched chain structure, the polarization distribution of the interface is optimized, and an efficient thermal conductivity network is constructed while suppressing the formation of charge transfer paths.

## 4. Conclusions

This article uses the method of molecular structure design to construct a new type of resin with micro-branched structure, and explores the influence of h-BN filling amount on the electrical and thermal properties of micro-branched modified BMI resin. At the same time, the BMI resin-based and micro-branched modified BMI resin-based thermally conductive composite materials are compared, revealing the regulatory mechanism of micro-branched structure on the electrical and thermal properties of thermally conductive composite materials. The thermal conductivity of BMI-DBA-TTMI/DMAA/h-BN increases with the increase in h-BN filling amount. When the filling rate is 40 wt%, the thermal conductivity is 1.51 W/(m·K). The micro-branched modified BMI resin promotes the wetting and permeation of thermally conductive fillers through the nanohole network of the branched skeleton in the molecular structure, optimizes the dispersion of fillers, and promotes the uniform distribution of thermally conductive fillers in the matrix, which is conducive to the construction of thermally conductive pathways. Compared with BMI-DBA/DMAA/h-BN, the thermal conductivity is increased by 15.27%. The insulation performance of composite materials increases first and then decreases with the increase in h-BN filling amount, and the insulation performance is at its most excellent when the filling amount is 10 wt%.

## Figures and Tables

**Table 1:** Parameters of the Y. Agari thermal conductivity model for composite materials.

Sample	C_1_	C_2_
BMI-DBA/DMAA/h-BN	1.192	0.489
BMI-DBA-TTMI/DMAA/h-BN	1.108	0.559

**Table 2:** The DMA data of BMI-DBA-TTMI/DMAA/h-BN.

Sample	^a^*T*_g_/℃	^b^*E*’_r_/GPa	^c^*ρ*/(mol·cm^−3^)
BMI-DBA-TTMI/DMAA/h-BN	512	5.91	0.463
10 wt%BMI-DBA-TTMI/DMAA/h-BN	505	5.33	0.323
20 wt%BMI-DBA-TTMI/DMAA/h-BN	504	4.88	0.298
30 wt%BMI-DBA-TTMI/DMAA/h-BN	499	3.98	0.295
40 wt%BMI-DBA-TTMI/DMAA/h-BN	497	2.12	0.291

**Table 3:** Weibull parameters of the AC breakdown strength of BMI-DBA-TTMI/DMAA/h-BN.

Sample	*E*_b_(kV/mm)	*β*
BMI-DBA-TTMI/DMAA/h-BN	35.62	22.09
10 wt%BMI-DBA-TTMI/DMAA/h-BN	36.28	18.85
20 wt%BMI-DBA-TTMI/DMAA/h-BN	32.41	15.26
30 wt%BMI-DBA-TTMI/DMAA/h-BN	30.70	17.36
40 wt%BMI-DBA-TTMI/DMAA/h-BN	28.46	16.31

**Table 4:** Activation energy of composites with different h-BN content.

Sample	Activation Energy (eV)
BMI-DBA-TTMI/DMAA/h-BN	0.236
10 wt%BMI-DBA-TTMI/DMAA/h-BN	0.219
20 wt%BMI-DBA-TTMI/DMAA/h-BN	0.409
30 wt%BMI-DBA-TTMI/DMAA/h-BN	0.498
40 wt%BMI-DBA-TTMI/DMAA/h-BN	0.581

**Table 5:** Weibull parameters of the AC breakdown strength of different materials.

Sample	*E*_b_(kV/mm)	*β*
BMI-DBA/DMAA/h-BN	24.04	17.13
BMI-DBA-TTMI/DMAA/h-BN	35.62	22.09
40 wt%BMI-DBA/DMAA/h-BN	18.73	13.25
40 wt%BMI-DBA-TTMI/DMAA/h-BN	28.46	16.31

**Table 6:** The activation energy of different materials.

Sample	Activation Energy (eV)
BMI-DBA/DMAA/h-BN	0.552
BMI-DBA-TTMI/DMAA/h-BN	0.236
40 wt%BMI-DBA/DMAA/h-BN	0.619
40 wt%BMI-DBA-TTMI/DMAA/h-BN	0.581

## Data Availability

The original contributions presented in this study are included in the article. Further inquiries can be directed to the corresponding authors.
